# Growth Differentiation Factor 15 in Severe Aortic Valve Stenosis: Relationship with Left Ventricular Remodeling and Frailty

**DOI:** 10.3390/jcm9092998

**Published:** 2020-09-17

**Authors:** Iacopo Fabiani, Tatiana Santoni, Marco Angelillis, Serena Petricciuolo, Andrea Colli, Giovanni Pellegrini, Deborah Mazzei, Nicola Riccardo Pugliese, Anna Sonia Petronio, Raffaele De Caterina

**Affiliations:** 1 Cardiac, Thoracic and Vascular Department, Pisa University Hospital and University of Pisa, Via Paradisa 2, 56124 Pisa, Italy; iacopofabiani@gmail.com (I.F.); angelillismarco@libero.it (M.A.); serena.petric84@yahoo.it (S.P.); colli.andrea.bcn@gmail.com (A.C.); as.petronio@gmail.com (A.S.P.); 2 Department of Surgical, Medical, Molecular Pathology and Critical Area, Pisa University Hospital and University of Pisa, Via Paradisa 2, 56124 Pisa, Italy; t.santoni@ao-pisa.toscana.it; 3 Clinical Chemistry Laboratory, Pisa University Hospital, 56124 Pisa, Italy; gn.pellegrini@ao-pisa.toscana.it (G.P.); d.mazzei@ao-pisa.toscana.it (D.M.); 4 Clinical and Experimental Medicine Department, University of Pisa, 56124 Pisa, Italy; n.r.pugliese88@gmail.com

**Keywords:** aortic valve stenosis, transcatheter aortic valve implantation, TAVI, frailty, biomarkers, growth/differentiation factor-15, GDF15, ventricular remodeling

## Abstract

**Background:** Frailty is an important outcome predictor in patients with aortic stenosis who are candidates for transcatheter or surgical aortic valve replacement (AVR). Growth/differentiation factor 15 (GDF15) is a cytokine playing a role in the pathophysiology of ventricular remodeling. We assessed its potential role as an independent soluble biomarker of frailty in these patients. **Methods:** We studied 62 patients (age, mean 79 years, 95% confidence interval (CI) 77–81; 54.8% female) with severe aortic valve stenosis and candidates for AVR. We systematically assessed pre-intervention GDF15 levels for their relationship with frailty (Katz score) and echocardiographic parameters of left ventricular dysfunction/remodeling. Fifteen hypertensive patients with left ventricular (LV) hypertrophy served as controls. **Results:** Patients with aortic valve stenosis featured higher GDF15 levels than controls (1773, 95% CI 1574–1971 pg/mL vs. 775, 95% CI 600–950 pg/mL, respectively, *p* < 0.0001). Subjects in the upper GDF15 tertile were older (*p* = 0.004), with a more advanced NYHA functional class (*p* = 0.04) and a higher prevalence of impaired renal function (*p* = 0.004). Such patients also showed a higher frailty score (*p* = 0.04) and higher indices of LV dysfunction, including reduced global longitudinal strain (*p* = 0.01) and a higher left ventricular mass (*p* = 0.001). GDF15 was significantly related to the Katz score, and predicted (OR 1.05; 95% CI 0.9–1.1; *p* = 0.03) a low (<5) Katz score, independent of the relationship with LV mass, age, renal function or indices of LV dysfunction. **Conclusions:** GDF15 is increased in patients with severe aortic stenosis and appears to be a soluble correlate of patients’ frailty, independent of indices of left ventricular dysfunction.

## 1. Introduction

Aortic valve stenosis (AVS) is the most frequent surgical indication for aortic valve replacement (AVR) in Western countries [1,2]. Transcatheter aortic valve implantation (TAVI) has emerged as a viable treatment of AVS in patients at high and intermediate surgical risk [1,2], and will soon be used more and more also in low-risk patients [3]. At present, a patient’s risk assessment is recommended before any treatment to orient clinical decisions, and is based on risk scores, such as the Euroscore or the Society of Thoracic Surgeons (STS) score [4]. Here, frailty has recently emerged as an important component of cardiovascular risk assessment, allowing for a more comprehensive patient characterization and risk stratification, taking into account subtle factors not included in conventional risk scores, and associates with short- and long-term mortality [5,6,7]. Due to this, the Valve Academic Research Consortium (VARC) has endorsed the integration of frailty tests in the heart team risk assessment before AVR [4]. 

One relevant research avenue and unmet need in the risk stratification of patients who are candidates for AVR is the possibility of integrating clinical and functional evaluations with information deriving from soluble biomarkers, which are easy to measure and add to current evaluations. Examples are cardiac troponins and natriuretic peptides, which have shown diagnostic and prognostic potential in such patients [8,9]. Several pre-clinical and clinical papers have demonstrated the pleiotropic role of growth differentiation factor 15 (GDF15) [10], a stress-responsive member of the transforming growth factor β (TGF-β) cytokine superfamily, in inflammation, immune-modulation, metabolism, cancer, metastatic shifting and neo-angiogenesis, as well as for the maintenance of tissue homeostasis and cellular stress response programs after injury [11]. GDF15 is synthesized as a 40-kDa pro-peptide with an N-terminal pro-peptide, and a C-terminal mature GDF15 domain. The dimeric precursor is then cleaved by pro-protein convertase to release a 24.5-kDa active circulating dimeric protein, which is secreted into the extracellular medium. Under resting conditions, limited production of GDF15 has been observed in the myocardium, but increased circulating GDF15 plasma concentrations have been linked to the development and progression of cardiovascular disease, and have been postulated to have a pathogenic (favorable or unfavorable) role [12], as well as to be potentially valuable prognostically [13], as has formerly emerged in mice models. GDF15 has been recently shown to be superior to natriuretic peptides for predicting risk in patients undergoing TAVI, and has incremental value over the logistic Euroscore [9]. Limited previous reports showed that GDF15 levels may reflect functional impairment associated with age and some frailty indices [14,15,16].

On this basis, we postulated a role of GDF15 in reflecting frailty in patients who are candidates for AVR. We therefore tested the hypothesis of an association between frailty and GDF15 levels, independent of myocardial structural or functional changes in patients who are candidates for AVR.

## 2. Methods

### 2.1. Study Population

Sixty-two patients with severe symptomatic AVS and preserved ejection fraction were prospectively enrolled at the Cardio-Thoracic and Vascular Department of Pisa University Hospital and screened between January and September 2016 for a prospective, echocardiographic, histopathology and circulating biomarkers correlation study to assess functional predictors of intervention outcomes [17]. Exclusion criteria were: age <18 years; presence of significant comorbidities other than AVS likely to affect prognosis (i.e., cancer; end-stage renal failure); an inadequate acoustic window; ischemic heart disease, including previous acute coronary syndromes; significant coronary artery disease defined as epicardial coronary artery disease >50% lumen diameter at coronary angiography; pregnancy; moderate or severe valvular disease in valves other than the aortic valve; the inability to sign consent; and the presence of septal dyskinesia (i.e., pacemaker-induced rhythm or intraventricular conduction disorders) precluding the evaluation of myocardial deformation by standard and 2D speckle tracking echocardiography (STE). The study protocol, at the time approved by the local ethics committee (ID No. 3726/2012), measured pre-intervention levels of high-sensitivity troponin T (TnT) and the N-terminal prohormone of brain natriuretic peptide (NT-proBNP), and allowed for the storage of plasma aliquots for further bio-humoral assessments. Written informed consent was obtained at the time also for future use of those samples. The present protocol, using aliquots from those plasmas and submitted to a new ethics committee approval (ID No. 14448, 14 May 2019), also included 15 healthy, stage I hypertensive subjects, age- and sex-matched with the patient cohort, with a recent (<3 months) echocardiographic examination judged to be within age-specific normal limits.

Patients with AVS underwent, at the time of the original study, the same laboratory tests, and an accurate trans-thoracic echocardiographic examination (TTE). The research was carried out according to the code of ethics of the World Medical Association (Declaration of Helsinki). Follow-up data were then obtained at 12 months from TAVI or AVR, at a scheduled outpatient cardiology visit, which included a follow-up echocardiographic examination.

### 2.2. Frailty Assessment

A frailty score was derived before intervention with the Katz index of independence of activities in daily living. This index, commonly referred to as the Katz ADL, is an appropriate instrument to assess functional status as a measurement of the patient’s ability to perform activities of daily living independently. We adopted the following definitions: >5 points = not frail; <5 points = frail [5,18,19].

### 2.3. Echocardiography

All patients underwent a complete transthoracic echocardiographic examination with a heart-dedicated machine (Vivid-E80, General Electric Milwaukee, WI, USA). Patients were studied in the left lateral decubitus, and data acquisition was performed with a matrix (M5S) probe at a depth of 16 cm in the apical (two-chamber, four-chamber and long axis) and parasternal (long- and short-axis) views according to current recommendations [20,21].

Using the standard M-mode and 2D images in the parasternal long-axis views, we calculated LV dimensions, including end-diastolic/end-systolic LV diameters, the end-diastolic thickness of the interventricular septum and the posterior wall. We used the body surface area (BSA) to correct left ventricular mass calculations and derive the LV mass index (LVMI). We used both the apical 2- and 4-chamber views to evaluate the LV end-diastolic and end-systolic volumes, and the standard Simpson’s rule to calculate the ejection fraction (EF). We performed spectral pulsed-wave Doppler analysis to assess LV diastolic function, measuring early (E-wave) and late (A-wave) trans-mitral velocities, the E/A ratio and the E-wave deceleration time (DT). We performed tissue Doppler imaging (TDI) assessments, adjusting the gain and frame rate to optimize tissue characterization. We used the continuity equation to calculate the indexed aortic valve area (AVAi), and the modified Bernoulli equation to estimate the maximum pressure gradient across the restricted orifice. We derived mean trans-aortic pressure gradients (MG) by averaging the instantaneous gradients over the ejection period measured by continuous-wave Doppler recordings. We also calculated the valvulo-arterial impedance (Z_VA_) as a measurement of global LV afterload. We used the color Doppler mode optimizing gain and the Nyquist limit to evaluate the presence of regurgitant valve disease. We assessed the severity of valvular regurgitation using a qualitative scale (mild, moderate or severe), according to current guidelines [20,21]. We used 2D speckle tracking echocardiography (2D STE) (EchoPAC Clinical Workstation Software v 12.0, GE Healthcare, frame rate 45-90 frames/s, fps) to asses LV global longitudinal strain (GLS), according to current standards [22]. Details on the methods used were previously reported [17].

### 2.4. GDF15 and Other Plasma Biomarkers

Preoperative GDF15 circulating levels (in pg/mL) were assessed on plasma samples from patients and control subjects with a sandwich immunoassay (ElectroChemiLuminescence ImmunoAssay, ECLIA—Elecsys, Roche, Basel, Switzerland), according to the manufacturer’s instructions. High-sensitivity Troponin T (hs-TnT STAT, Roche, Basel, Switzerland) and N-terminal pro-brain natriuretic peptide (proBNP II STAT, Roche, Basel, Switzerland) were also assessed. Renal function (estimated glomerular filtration rate, eGFR) was estimated with the Modification of Diet in Renal Disease (MDRD) formula. Biomarkers levels were normalized (log_10_) for statistical analysis when appropriate.

### 2.5. Statistical Analyses

We used the Kolmogorov–Smirnov test to assess the possible deviation from the normality of the data distribution. Continuous variables are presented as mean and 95% confidence intervals (CI) if normally distributed, or as median and 95% CI otherwise. We reported categorical data as percentages. Circulating biomarker levels were treated as continuous variables or were normalized (as to natriuretic peptides) with log transformation. We compared continuous variables between patients and controls using the Student’s t-test in the case of normal, or the Mann–Whitney U-test in the case of non-normal distributions. We assessed the correlation (r coefficients) when appropriate. We used the analysis of variance (ANOVA) or the Kruskal–Wallis test to evaluate the distribution of circulating biomarker levels in different classification categories, with proper post hoc corrections for interactions. For discrimination of patients with high frailty according to the Katz score, we used the c-statistic to derive the GDF15 value with the best combination of sensitivity and specificity. We built a logistic regression model, with the Katz score as a dependent, dichotomic (<5 or ≥5) variable. We used multivariable, stepwise regression analysis to identify predictors of reverse remodeling after the evaluation of collinearity. In the text, we considered the global longitudinal strain (GLS) in absolute values to preserve the statistical meaning of regression analysis (direct/inverse correlation/association). We set the threshold for statistical significance at *p* < 0.05. For all calculations, we used the Medcalc 12.7 (Medcalc Softwares, 2013, city, Belgium) statistical package.

## 3. Results

The study population included 62 patients (median 79.3 years, 95% CI 77.3–81.3 years; of whom *n* = 34 (55%) were female) and 15 matched healthy stage-I hypertensive controls (78.2 years, 95% CI 76.5–80.2 years; of whom 8 (55%) were female; for all comparisons, *p* = N.S.). Demographic, bio-humoral and echocardiographic characteristics of the overall population with aortic stenosis, divided into tertiles 1 and 2 combined vs. tertile 3, are summarized in Table 1 and Table 2, respectively.

**Figure 1:**
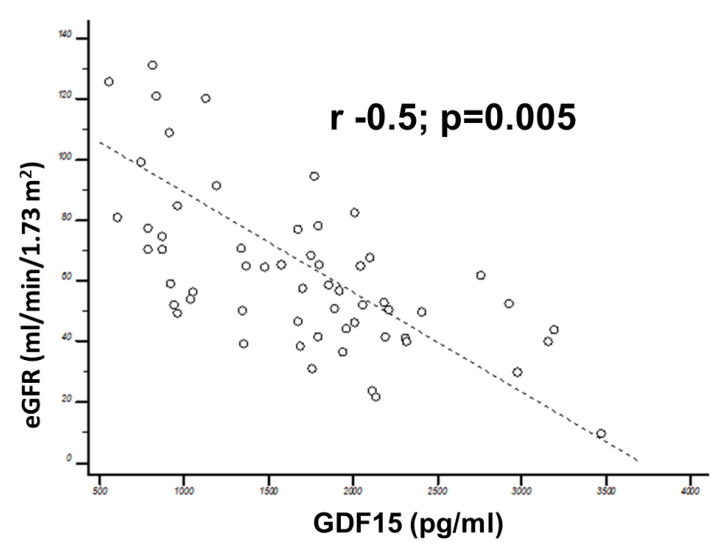
Correlation between Growth differentiation factor 15 (GDF15) and estimated glomerular filtration rate. Note the significant inverse relationship between renal function and GDF-15 levels.

Patients with AVS had higher hs-TnT (89.5 ng/L, 95% CI 20.4–110.7 vs. 4.8 ng/L 95% CI 4–5.6; *p* = 0.04), NT-pro-BNP (918 ng/L, 95% CI 588.2–1433.6 vs. 33.7 ng/L 95% CI 16.7–50.6; *p* = 0.0001) and GDF15 levels (1772.4 pg/mL, 95% CI 1573.5–1971.3 vs. 774.9 pg/mL 95% CI 599.5–950.2; *p* < 0.0001) compared to controls. They also featured more impaired renal function (eGFR 63.3 mL/min/1.73 m^2^, 95% CI 56.6–70 vs. 73.3 mL/min/1.73 m^2^, 95% CI 68.8–75.4; *p* < 0.0001). There were no differences in GDF15 levels across sexes (male sex: 1525.4 pg/mL, 95% CI 1254.1–1716.6 vs. 1624.2 pg/mL, 95% CI 1361.5–1886.8; *p* = 0.7), while we found an inverse association with renal function (eGFR: r = −0.5; *p* = 0.005; Figure 1). GDF15 levels correlated with hs-TnT (r = 0.5; *p* = 0.03) and NT-pro-BNP levels (r = 0.64; *p* < 0.0001). By dividing the aortic stenosis population into tertiles of GDF15, we found that patients in the upper GDF15 tertile were older (81 years, 95% CI 79.4–82.5 vs. 76.2 yrs, 95% CI 73.3–79.2; *p* = 0.004), with a more advanced New York Heart Association (NYHA) functional class (NYHA III 17, 43.6% vs. 7, 30%; *p* = 0.04), more impaired renal function (eGFR 54.2 mL/min/1.73 m^2^, 95% CI 47.6–60.9 vs. 69.4 mL/min/1.73 m^2^, 95% CI 62.2–73.6; *p* = 0.004) and higher biomarkers levels (hs-TnT 54.1 ng/L, 95% CI 49.5–98.8 vs. 24.8 ng/L, 95% CI 4.8–44.8; *p* = 0.0001 and NT-pro-BNP 1048.5 ng/L, 95% CI 510.2–1371.4 vs. 140 ng/L, 95% CI 78–251.2; *p* < 0.0001).

### 3.1. Correlation of GDF15 with Echocardiographic Data

In patients with AVS, GDF15 levels correlated with left atrial volume (r = 0.34; *p* = 0.01) and pulmonary artery systolic pressure (PAPs, r = 0.36; *p* = 0.01). In particular, subjects with a significantly dilated left atrium (indexed left atrial volume, LAVi > 34 mL/m^2^) had higher GDF15 levels (*p* = 0.002). Patients in the upper GDF15 tertile also featured a reduced global longitudinal strain (GLS, 13.9%, 95% CI 12.7–15.1 vs. 15.9%, 95% CI 14.7–17.2; *p* = 0.01) and a higher indexed left ventricular mass (LVMi, 132.5 g/m^2^, 95% CI 124.3–140.7 vs. 111.4 g/m^2^, 101.2–121.6; *p* = 0.001).

### 3.2. Correlations with Frailty

**Figure 2:**
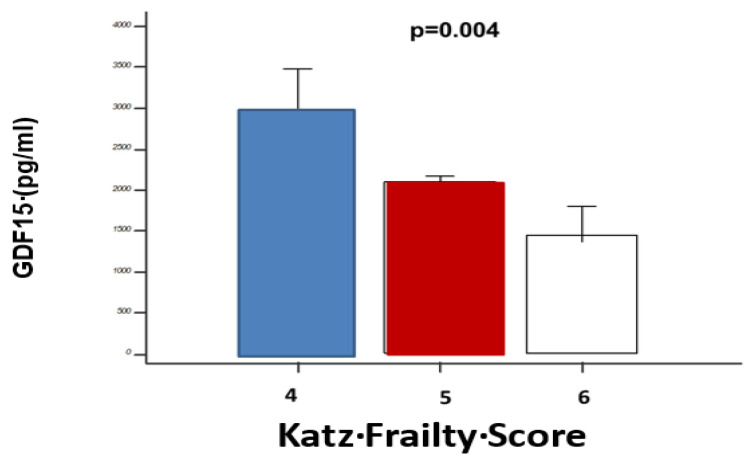
Relationship of GDF15 levels with the Katz frailty score. Analysis of variance shows significant differences in circulating GDF15 levels (pg/mL) as a function of the Katz frailty score (4 to 6), with higher values in patients with a lower Katz score (higher frailty).

The Katz score showed low frailty (Katz ≥ 5 in *n* = 52 patients, 83.9%) in the majority of AVS patients who are candidates for AVR (5 and 6 points: 14, 22.6% and 38, 61.3% patients, respectively), with a low prevalence of higher frailty grades (Katz < 5, and specifically, 4 points in 10 (16.1%) patients). Related to the circulating biomarkers here explored, we found a statistically significant difference in the distribution as per the Katz score (4 to 6 points) only for GDF15 (for hs-TnT *p* = 0.1; NT-pro-BNP *p* = 0.08), with higher levels reported for progressively higher frailty grades (*p* = 0.04) (Figure 2), as reflected by a differential distribution according to the tertiles of GDF15 (9 (23%) of Katz < 5 in the upper tertile vs. 1 (4.3%) in the lower ones; *p* = 0.0001)(see also Table 1).

**Figure 3:**
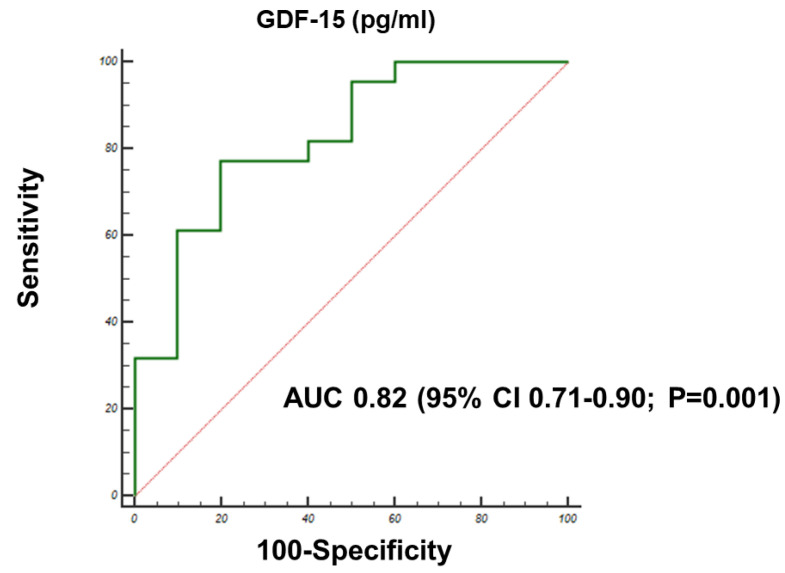
Receiver-operator curve and c-statistic analysis for the ability of GDF15 plasma levels > 2500 pg/mL to predict frailty (Katz score < 5). GDF15 levels > 2500 pg/mL had the highest AUC for discriminating more frail patients.

At the receiver-operator curve (ROC) analysis to assess the specificity and sensitivity of GDF15 for predicting frailty, a GDF15 level >2500 pg/mL had the highest area under the curve (AUC = 0.82, 95% CI 0.71–0.90; *p* = 0.001) for discriminating more frail patients (i.e., Katz score < 5) (Figure 3). No relevant discriminative power in terms of frailty was found for the other circulating biomarkers included in the study.

At the multivariable stepwise logistic regression analysis, including only variables correlating with a normal (≥5) Katz score at the univariable analysis—GLS, age, eGFR, NYHA functional class, LVMi and GDF15—GDF15 ended up to be the only significant predictor of frailty (Katz score < 5) (Table 3).

### 3.3. Correlations with Follow-Up Outcomes

Thirty patients underwent AVR (of whom 25 (83%) with a surgically implanted bioprosthesis). The other 32 patients (51.6%) underwent TAVI. Patients selected for TAVI, as per Euroscore II (5.3% vs. 2.3%; *p* < 0.0001), had, on average, a higher NYHA functional class (NYHA III: 18, 56.2% vs. 6, 20%; *p* = 0.04) and a higher frailty score (Katz score < 5, *n* = 9, 28%; vs. *n* = 1, 3.4%, *p* < 0.0001). These patients also had more impaired renal function (eGFR, mL/min/1.73 m^2^, 53.6, 95% CI 46.5–60.8 vs. 73.3, 95% CI 62.7–84; *p* = 0.02), and higher levels of circulating biomarkers (NT-pro-BNP, ng/L: 1084.9, 95% CI 541.3–1430.6 vs. 349.8, 95% CI 205.1–596.7; *p* = 0.0001; hs-TnT, ng/L: 66.7, 95% CI 33.8–99.7 vs. 14.5, 95% CI 10.3–18.7; *p* = 0.004). These patients also had higher levels of GDF15, pg/mL: 1977.3, 95% CI 1697.3–2257.3 vs. 1522.1, 95% CI 1233.2–1811; *p* = 0.02. They did not, however, differ as to age and echocardiographic variables. After an average follow-up of 11 ± 3 months, neither major procedural complications nor long-term cardiovascular events were noted.

## 4. Discussion

In the present study, conducted in a population of patients with severe AVS who are candidates for AVR or TAVI and in age- and sex-matched controls, we found:

- Higher GDF15 levels in AVS patients compared to age- and sex-matched controls;
- An association between age, NYHA functional class, renal function and GDF15 levels;
- A differential distribution of GDF15 levels according to parameters of systolic function (GLS) and structure (LAVi; LV mass);
- A differential distribution of GDF15 according to the frailty score (Katz index), with higher GDF15 levels in more frail patients, independent of other clinical, structural and bio-humoral parameters.

Of novelty, we thus propose GDF15 as a soluble biomarker reflecting structural and functional alterations, as well as—and independent of them—an indicator of frailty in patients who are candidates for treatment. While we could not show, likely due to the limited sample size, a relationship with prognosis, our pilot study should prompt further investigations on the possible use of this biomarker in such patients.

### 4.1. GDF15 in Aortic Valve Disease

GDF15 [10] is a pleiotropic cytokine, with proved effects on inflammation, tumorigenesis, metastatic shifting and metabolism [23] and maintenance of tissue homeostasis, and expressed as part of the stress response program of cells after cellular injury in pre-clinical and clinical contexts [10]. GDF15 has been detected in the heart, intestine, liver, kidney, pancreas, colon, brain and skeletal muscle. In the cardiovascular field, GDF15 has been postulated to play a role in the pathophysiology of LV remodeling, hypertrophy and myocardial fibrosis, with substantial implications in heart failure [24,25]. A direct correlation between LV mass and GDF15 levels in a context of hypertrophy linked to AVS, heart failure or arterial hypertension had already been reported [26,27,28], also accounting for the increasing fibrosis grade observed with aortic valve disease progression.

A translation of this information to the broad setting of risk evaluation in patients with AVS who are candidates for an intervention was logical. Here, the concept of risk stratification using biomarkers is appealing [8,17,29,30]. Recently, GDF15 has been shown to be superior to natriuretic peptides for predicting risk in patients undergoing TAVI, and has been shown to have incremental value over the logistic Euroscore, enabling a substantial reclassification of patients [9,31]. GDF15 levels were indeed significantly associated with procedural variables and with reduced kidney function (*p* = 0.001), high creatinine (*p* = 0.002) and high NT-proBNP levels (*p* < 0.001) [31]. These previous studies thus promoted the idea of GDF15 as a useful addition in patient assessment for TAVI. Moreover, Kim et al. showed that GDF-15 is associated with the absence of ventricular recovery (>20% LV mass reduction) after TAVI [26]. Similar to those findings, AVS patients in our study showed increased GDF15 levels compared to controls, and these correlated with age, renal function and NYHA functional class. A previous pilot paper [32] on 22 patients undergoing AVR showed a preoperative mean plasma GDF15 level similar to healthy controls, with an increase in plasma concentrations at two days postoperatively and a subsequent decline at 6 and 12 months postoperatively. In that study, GDF15 correlated significantly with the peak aortic gradient at six months postoperatively, while it did not correlate with LVMi at any time point throughout the one-year observation period. Contrary to that study, we here found a differential distribution of GDF15 levels according to LV structural (including LAVi and baseline LVMi) and systolic function parameters (GLS), thus arguing for a potential link between this biomarker and changes in ventricular structure and function. The larger sample size of our population compared with the previous report [32] may be the reason for the different findings. Based on our data, preoperative GDF15 could be proposed as a marker of adverse cardiac remodeling, possibly refining decisions about the timing of valve replacement.

### 4.2. GDF15 and Frailty Before Aortic Valve Replacement

AVR is the gold standard of treatment for severe AVS [1,33], and TAVI is currently mostly recommended for patients at higher surgical risk. Guidelines always insist on a multidisciplinary collaborative agreement and on a joint decision process to ensure the appropriate patient selection for the optimal timing and technique of AVR [1,33]. Besides anatomical considerations, and evaluations of comorbidities and of surgical risk, an assessment of frailty has become pivotal, in recent years, for choosing the right treatment option in each patient [1,2]. There is, however, the lack of a standard, consistent definition of frailty. In an expert consensus for physical frailty published in 2013, the authors included wasting, loss of endurance, decreased balance and mobility, slowed performance, relative inactivity and decreased cognitive function as indicators of a frailty condition [34]. Among other indicators, the Katz index is used for the functional assessment of dependency in elderly individuals in the six domains of feeding, bathing, dressing, transferring, toileting and urinary continence [18]. The index thus provides objective data on the patient’s independence in daily activities. The Katz score dichotomically classifies (yes or no) each item of independence in the six above-listed domains. The test has demonstrated its utility in evaluating functional status in the elderly population [35]. Specifically to AVR, Puls et al. identified a reduced Katz index as a significant independent predictor of immediate, short- and long-term all-cause mortality [6]. Similarly, other studies [36,37] have included the Katz score in their combined frailty indices. In a study by Green et al., subjects with physical limitations, as defined by the Katz score, showed a trend towards higher one-year mortality compared to patients without limitations [38]. Recent reports have confirmed the improvement of clinical prediction models by the addition of frailty scores [39,40]. A recent study by Rogers et al. on 544 consecutive TAVI patients reported an association between frailty status (Katz score ≤4) and mortality, incrementally improving the STS risk prediction model [41]. However, evaluation of frailty may itself benefit from additional, objective (i.e., quantifiable) parameters, reflecting a global, systemic burden of deterioration. Recent recommendations mention anemia and serum albumin as the most useful biochemical parameters to stratify frailty status [42].

Here, we report, for the first time, an independent association between GDF15 and frailty as assessed with the Katz score, after weighting the association for structural, functional or other bio-humoral parameters. Previous reports have shown that GDF15 levels reflect progressive patients’ functional impairment (including muscle wasting) and renal dysfunction, being associated with both aging and, independently, with frailty indices (not including the Katz Score) [14,15,16]. Our report therefore proposes the possible usefulness of this new biomarker in the realm of AVR.

The cut-off point for the Katz score of 4 we used to define frailty in our study is similar to what has been used in previous studies showing a significant prognostic impact [9]. Moreover, GDF15 was the only circulating biomarker associated with frailty in our study, resulting higher in the TAVI group, conventionally including patients at higher risk, potentially in a later stage of disease. We propose the maintenance of this cut-off for practical translation in the evaluation of patients who are candidates for AVR.

Mechanisms for the elevation of GDF15 in frail patients with AVS are speculative, and out of the scope of this report. We may speculate that GDF15 captures the realm of inflammation, altered metabolism or impaired homeostasis. This was not the case for NT-pro-BNP and hs-TnT in our population. As AVS is a slowly progressive, chronic disease, similar in this respect to cancer, the application of a holistic view with the inclusion of biomarkers, as is currently done in oncology, could potentially be helpful in patient risk stratification [43].

## 5. Limitations

Due to the low number of events here accrued, we could not draw prognostic conclusions. Previous findings showed that plasma levels of GDF15 were significantly elevated after 5 and 20 min of myocardial reperfusion after AVR [44]. We did not assess postoperative GDF15 levels to evaluate the impact of such changes on remodeling or prognosis.

A second limitation is that we did not assess other frailty indices, but relied on the single, time-honored, highly recognized Katz score.

Further, we included a mixed population of surgically and percutaneously treated AVS patients, thus reflecting the relative heterogeneity of the Katz score and the low number of significantly frail (i.e., Katz’s score < 5; *n* = 10) patients, as previously shown [8].

A further limitation of our study is the limited ability to infer reasons or mechanisms for the GDF15 increase in our series. GDF15 may reflect a systemic deterioration and metabolic alteration: thus, it may be non-specific for the population with AVS: patients here included were indeed substantially free from other severe comorbidities (e.g., cancer, immune disorders, infections) and only selected for having an indication for AVR, precluding an analysis of the specificity of our findings. Our multivariable analysis took into account age and renal function, but we found no difference according to diabetic or hypertensive status.

## 6. Conclusions

GDF15 levels are increased in patients with AVS. This may indicate functional and structural ventricular changes induced by progressive pressure overload, with potential implications in terms of reverse remodeling. GDF15 circulating levels may reflect the systemic conditions underlying a frail patient phenotype, in addition to comorbidities. Such a role for this cytokine should be re-evaluated in a larger prospective patient cohort.

## Figures and Tables

**Table 1:** Demographic and biohumoral characteristics of the study population (whole population and according to GDF15 tertiles, T) and controls.

	Population (*n* = 62)		Controls (*n* = 15)			GDF15 T1-T2 (754–1643 ng/L) (*n* = 23)		GDF15 T3 (2255–2725 ng/L) (*n* = 39)		T1-T2 vs. T3
	N (Median/Mean)	% (95% CI)	N (Median/Mean)	% (95% CI)	*p* *	N (Median/Mean)	% (95% CI)	N (Median/Mean)	% (95% CI)	*p*
Gender (Female)	34	54.8%	8	55%	ns	12	52.1%	22	56.4%	0.5
Age (years)	79.3	77.3–81.3	78.2	76.5–80.2	ns	76.2	73.3–79.2	81	79.4–82.5	0.004
BMI (kg/m^2^)	26.6	25.6–27.6	25.5	25.2–27.7	ns	26.6	25–28.2	26.6	25.2–27.9	0.9
BSA (m^2^)	1.8	1.81–1.95	1.75	1.71–1.96	ns	1.88	1.8–1.96	1.81	1.75–1.87	0.2
Heart Rate (bpm)	74	69.3–78.7	71	68.1–79.6	ns	72.1	67.9–76.3	77.3	73–81.7	0.1
Atrial Fibrillation	6	9.6%	0	0%	NA	1	4.3%	5	12.8%	0.06
SAP (mmHg)	140.2	132.9–147.5	140.2	132.9–147.5	ns	135.6	128.9–142.3	140.2	133.2–147.2	0.4
DAP (mmHg)	71.1	67.2–74.9	71.8	67–75.3	ns	71	66.9–75.1	71.3	67.1–75.4	0.9
Smokers	17	27.4%	0	0%	NA	7	30.4%	10	25.6%	0.07
Anemia	20	32.2%	0	0%	NA	6	26%	14	35.8%	0.08
Diabetes Mellitus	17	27.4%	0	0%	NA	7	30.4%	10	25.6%	0.07
Hypertension	57	92%	13.5	90%	ns	21	91.3%	36	92.3%	0.07
Coronary Artery Disease	19	30.6%	0	0%	NA	7	30%	12	30.7%	0.6
COPD	15	24.1%	0	0%	NA	4	17.3%	11	28.2%	0.06
NYHA class I/II	38	61.2%	15	100%	<0.0001	20	86.9%	18	46.1%	0.03
NYHA class III	24	38.7%	0	0%	NA	7	30%	17	43.6%	0.04
Katz Frailty Score ≥5	52	83.8%	NA	NA	NA	22	95.6%	30	77%	0.05
Katz Frailty Score <5	10	16.1%	NA	NA	NA	1	4.3%	9	23%	0.0001
eGFR (mL/min/1.73 m^2^)	63.3	56.6–70	73.3	68.8–75.4	<0.0001	69.4	62.2–73.6	54.2	47.6–60.9	0.0001
Euroscore II (%)	3.8	2.7–4.9	NA	NA	NA	3.4	1.2–5.7	4	2.9–5.2	0.6
GDF-15 (pg/mL)	1772.4	1573.5–1971.3	774.9	599.5–950.2	<0.0001	1253.8	1115.5–1392.2	2490.4	2255–2725.7	<0.0001
NT-pro-BNP (ng/L)	918	588.2–1433.6	33.7	16.7–50.6	0.0001	140	78–251.2	1048.5	510.2–1371.4	<0.0001
TnT-Hs (ng/L)	89.5	20.4–110.7	4.8	4–5.6	0.04	24.8	4.8–44.8	54.1	49.5–98.8	0.0001
Albumin (g/dl)	3.8	3–4.4	4.2	4–4.6	ns	3.5	2.9–3.8	3.6	3.1–4.1	0.8

Data are presented in numbers (%) or Mean/median (95% CI); * vs. whole population. BMI: body mass index; BSA: body surface area; CAD: coronary artery disease; COPD: chronic obstructive pulmonary disease; DAP: diastolic arterial pressure; eGFR: estimated glomerular filtration rate; GDF-15: Growth-differentiation-factor-15; NT-pro-BNP: N-terminal-pro-brain natriuretic peptide; NA: Not assessed; NYHA: New York Heart Association; SAP: systolic arterial pressure; TnT-Hs: High sensitivity Troponin-T; TAVI: trans-catheter aortic valve implantation.

**Table 2:** Echocardiographic parameters (whole population, controls, and GDF15 tertiles (T)).

	Population (*n* = 62)		Controls (*n* = 15)			GDF15 T1-T2 (754-1643 ng/L) (*n* = 23)		GDF15 T3 (2255-2725 ng/L) (*n* = 39)		
	N (Median/Mean)	% (95% CI)	N (Median/Mean)	% (95% CI)	*p* *	N (Median/Mean)	% (95% CI)	N (Median/Mean)	% (95% CI)	*p*
LV EDVi (mL/m^2^)	53	48.6–57.4	56	49.6–59.4	ns	50	44.3–56.9	54.3	48.3–60.4	0.4
LV ESVi (mL/m^2^)	28.1	24.3–31.9	26.1	23.3–33.9	ns	26.9	22.3–31.6	28.8	23.2–34.3	0.6
EF (%)	60.7	57.5–63.8	62.7	58.5–65.8	ns	62.7	59.9–65.4	59.9	56.6–63.3	0.2
GLS (%)	13.9	12.9–15	17.2	15.9–19	<0.0001	15.9	14.7–17.2	13.7	12.7–15.1	0.01
RWT	0.50	0.47–0.53	0.38	0.35–0.42	<0.0001	0.5	0.46–0.53	0.51	0.48–0.54	0.5
LVMi (g/m^2^)	130.1	119.6–140.5	80.2	65.4–88.3	<0.0001	111.4	101.2–121.6	132.5	124.3–140.7	0.001
LAVi (cm^2^/m^2^)	44.1	37–47	26	22.2–31.1	<0.0001	41.7	35.1–46.4	47.3	41.3–50.2	0.04
E/A	0.8	0.6–0.9	1	0.8–1.2	ns	0.7	0.6–0.8	0.8	0.7–0.9	0.8
E/e’average	15.8	13.1–17.9	11.5	9.1–13.2	0.04	16.3	13.4–19.1	16.9	14.4–19.4	0.7
Stroke Volume index (mL/m^2^)	35	31.4–38.6	49	45–65	<0.0001	36.7	31.9–41.5	33.4	30.2–36.5	0.2
AVAi (mL/m^2^)	0.36	0.34–0.4	NA	NA	NA	0.4	0.35–0.43	0.38	0.33–0.41	0.4
Peak Trans-aortic velocity (m/s)	4.4	4.2–4.6	1.6	1.5–2.1	<0.0001	4.4	4.2–4.7	4.3	4.2–4.5	0.4
Maximum pressure gradient (mmHg)	79.9	73.6–86.3	NA	NA	NA	82.5	73.8–91.1	77.5	73.1–82.4	0.3
Mean pressure gradient (mmHg)	49.7	45.7–53.8	NA	NA	NA	50.3	44.7–55.9	47.7	44.6–50.7	0.4
ZVa (mmHg/mL/m^2^)	5.4	4.9–5.8	NA	NA	NA	5.2	4.5–5.8	5.4	4.9–5.8	0.6
Estimated sPAP	33.1	29.8–32.4	28.8	27.9–31.3	0.03	30.5	27.9–33	33.9	30.1–37.7	0.2
TAPSE (mm)	19	16.7–24	20	18.3–25.2	0.04	18	17.5–23.3	17	16.8–22.8	0.6

Data are presented in numbers (%) or mean/median (95% CI); * vs. whole population. AVAi: aortic valve area index; E/A: ratio of early to late diastolic mitral filing velocity; EDVi: indexed end-diastolic volume; E/e’: ratio of trans-mitral early diastolic velocity to tissue early diastolic mitral annular velocity (TDI); EF: ejection fraction; ESVi: end-systolic volume indexed; GLS: global longitudinal strain; LAVi: indexed left atrium volume; LVMi: indexed left ventricular mass; LV: left ventricle; NA: Not assessed; RWT: relative wall thickness; sPAP systolic pulmonary artery pressure; TAPSE: tricuspid annular plane systolic excursion; ZVA: valvulo-arterial impedance.

**Table 3:** Logistic uni- and multi-variable regression analysis with a Katz score <5 as the dependent variable.

Independent Variables	OR	95% CI	*p*	OR	95%CI	*p*
**GLS%**	1.062	1.002–1.112	0.04			0.07
**Age (years)**	1.065	1.002–1.114	0.05			0.08
**GDF-15 (pg/mL)**	1.2	1001–1.324	0.02	1.06	1–1.123	0.03
**LVMi (g/m^2^)**	0.9	0.8–0.987	0.04			0.1
**NYHA class**	0.85	0.78–0.943	0.03			0.2
**eGFR (mL/min/1.73 m^2^)**	1.12	1.004–1.253	0.038			0.09

eGFR: estimated glomerular filtration rate; GLS: global longitudinal strain; GDF-15: Growth-differentiation-factor-15; LVMi: left ventricular mass indexed; NYHA: New York Heart Association.
